# Establishment of Hospitals in China

**Published:** 1841-07

**Authors:** 


### ESTABLISHMENT OF HOSPITALS IN CHINA.

In an early number of this Journal, (vol. IV. p. 568,) we noticed the establishment of Medical Missions in China; and we are much gratified to learn that they have been most successful in their double capacity of providing for the temporal and spiritual wants of the singular people on the borders of whose vast country they are planted. One of the most zealous and enlightened of these excellent missionaries, the Rev. Peter Parker, M.D. of the United States, is now in England for the purpose of raising "a permanent fund for the support of the 'Medical Missionary Society in China,' for the maintenance of the hospitals already established, and for the founding of others at every accessible and eligible part of China." The efforts to benefit the Chinese in this way in modern times are, we are told, briefly these. Alexander Pierson, Esq. surgeon to the Honorable East India Company, introduced successfully the art of vaccination in 1805; this has since extended widely through the empire. Dr. Livingston and Rev. Dr. Morrison opened an infirmary for the poor Chinese at Macao in 1820, which was sustained for some time, and alleviated much suffering. In 1827, T. R. Colledge, Esq. surgeon to the Honorable East India Company, opened his Eye Infirmary at Macao, and, during the three years of its continuance, afforded relief to no less than 4000 patients, among whom were persons in different ranks, and from various parts of the empire, from whom he received many and unequivocal tokens of gratitude. The Ophthalmic Hospital at Canton was opened by the Rev. P. Parker, M.D., October, 1835, and the General Hospital at Macao, in July, 1838. Up to the 17th June, 1840, these institutions had received upwards of 8000 patients, embracing every variety of disease. It was after long effort that a place was found for an hospital; and when at length a suitable building was rented, and previous notice had been given, the first day no patients ventured to come; the second, a solitary female affected with glaucoma came; the third day, half a dozen; and soon they came in crowds. Since then patients from all parts of the empire have availed themselves of the benefits of the hospital; persons of all ranks, military, naval, and civil officers, the Nanhæhêer, or district magistrate, the custom-house officer, salt inspectors, provincial judges, provincial treasurer, a Tartar general, governors of provinces, Commissioner Lin himself, and even a member of the imperial family.

The following extract from the same pamphlet which has supplied us with the above information respecting hospitals in China,* and which we recommend to the notice of our readers, points out, in a few words, the nature of the noble cause in which this gentleman is engaged, and which cannot fail to commend itself to every philanthropist.

"That the union of the art of healing with that of teaching, in the missionary of modern times, is as important as in the early ages of Christianity, is no longer doubtful. The experiment has been made, and succeeds. Healing by miraculous agency was employed at the commencement of the Christian era, chiefly as other preternatural powers were to establish the divinity of Christianity. A still further object was to exhibit the beneficial spirit of the gospel. The age of miracles and the occasion for them ceased together; but the *spirit* of the gospel is the same in every age. Healing the sick, opening the eyes of the blind, and the ears of the deaf, and causing the tongue of the dumb to speak, and the lame to walk, by natural and scientific means, is not less calculated in the nature of things, to conciliate favour, and to demonstrate the disinterested and benevolent genius of Christianity now, than it was eighteen centuries ago. Though the practice of medicine and surgery among western nations is founded upon *science*, yet, to an uncivilized and superstitious nation, it has much of the appearance of a superhuman power, which may lawfully subserve a good end, if the truth of the case be distinctly stated, and their credulity be not imposed upon. The gratuitous practice of medicine and surgery, founding hospitals

* "Statements respecting Hospitals in China," by the Rev. Peter Parker, M.D.—London, 1841.

and infirmaries, confer a direct and great good upon suffering humanity. This species of charity is peculiarly needed in China. To sustain the hospitals already established in the empire, and to multiply them as the way is prepared, and in them to train up Chinese youth, to extend the blessings beyond the limits to which the policy of the government restricts foreigners, and to give a correct and scientific practice of medicine and surgery to an empire which exceeds in territory and population any other nation, is of itself a grand enterprise. The undertaking so great, is also *practicable.* Had the object no claims beyond those already alluded to, it would be deemed sufficient in respect to any christianised country; but in relation to China, they are the subordinate claims, compared with still higher ones, to which they conduct. In exhibiting the utility and importance of this object, let it not be supposed that any other is displaced. It is not to be lost sight of for a moment, that *Divine truth* is the great agent through which our ultimate aim is to be gained. While by the needle of the oculist the light may be poured upon the eye long dark, by the surgeon's knife the useless limb amputated, and by the physician's skill even the malignant disease may be cured, nothing short of a higher power can, in a moral sense, remove the film from the eye, clarify the spiritual vision, and heal the malady of sin. At present, however, it is but to a limited degree that the higher means can be employed; but to exhibit the spirit and the fruits of the gospel, Providence has remarkably opened the way; and it is fondly hoped that at no distant day, the Chinese will regard these benevolent offices as such, and permit us to publish and enforce the *precepts* of Christianity. After several years' experience and residence in China, the firm belief is expressed, that in the present state of the Chinese, who are prejudiced against foreigners, as avaricious and barbarous, and possessed of no redeeming qualities, there is no method so directly adapted to remove false impressions, and to convince them of the true character of Christian men and the Christian religion, as by the plan adopted by the Medical Missionary Society in China."

We earnestly commend the sacred cause in which Dr. Parker and his brethren are engaged, to the attention of all whose position and means enable them to promote it. Independently of the richer fruits of their heroic labours, we look forward, with confidence, to great benefits which medicine may expect from them, in the observation of new forms of disease, and in the discovery of new therapeutic means in the natural productions of this vast and unknown country, and amid the mountains of pharmaceutic rubbish which have been accumulating in their traditionary and written records, from periods anterior to all occidental history. We shall even hope for no inconsiderable addition to our stock of oriental medicine, from the work which Dr. Parker is now preparing for the press, founded on his observation and practice in China.

---

